# Economic evaluation of artesunate and three quinine regimens in the treatment of severe malaria in children at the Ebolowa Regional Hospital-Cameroon: a cost analysis

**DOI:** 10.1186/s12936-016-1639-1

**Published:** 2016-12-07

**Authors:** Daniel Ethe Maka, Andreas Chiabi, Bolaji Obadeyi, Evelyn Mah, Séraphin Nguefack, Pamela Nana, Wilfred Mbacham, Elie Mbonda

**Affiliations:** 1 Department of Paediatrics, Faculty of Medicine and Biomedical Sciences, University of Yaounde I, Yaoundé, Cameroon; 2 Paediatric Unit, Yaounde Gynaeco-Obstetric and Paediatric Hospital, Yaoundé, Cameroon; 3 Healthlogics Limited, Lagos, Nigeria; 4 Paediatric Unit, Ebolowa Regional Hospital, Ebolowa, Cameroon; 5 Department of Physiology and Biochemistry, Faculty of Medicine and Biomedical Sciences, University of Yaoundé I, Yaoundé, Cameroon

**Keywords:** Artesunate, Quinine, Cost, Cost-analysis, Severe malaria, Children, Cameroon

## Abstract

**Background:**

Severe malaria is a leading cause of morbidity and mortality in under-fives in sub-Saharan Africa. Recently quinine has been replaced by artesunate as the first-line drug in the treatment of severe malaria in Cameroon. Artesunate has been shown to be cost-effective in African children, but whether these findings are transferable to Cameroonian children remains to be explored.

**Objectives:**

To conduct a cost-analysis of four different regimens used in the treatment from the perspective of the healthcare payer.

**Methods:**

An economic evaluation alongside a randomized comparative study was conducted in children aged 3 months to 15 years, admitted at the Ebolowa Regional Hospital with severe malaria due to *Plasmodium falciparum.* Patients were randomized to receive one of the four treatment alternatives. Group 1 (ARTES) received parenteral artesunate at 2.4 mg/kg at H_0_, H_12_, H_24_ and then once daily; Group 2 (QLD) received a loading dose of quinine base at 16.6 mg/kg followed 8 h later by an 8-hourly maintenance dose of 8.3 mg/kg quinine base; Group 3 (QNLD3) received 8.3 mg/kg quinine base every 8 h, and Group 4 (QNLD2) received 12.5 mg/kg quinine base every 12 h. The main outcome measure for effectiveness of treatment was the parasite reduction rate. Based on a healthcare perspective, an evaluation of direct medical costs was done, including costs of anti-malarials, nursing care materials, adjuvant treatment, laboratory investigations, hospitalisation and professional fees. Guided by a cost minimalization approach, the relative costs of these treatment alternatives was compared and reported.

**Results:**

Overall cost was higher for ARTES group at $65.14 (95% CI $57.68–72.60) than for quinine groups ($52.49–$62.40), but the difference was not statistically significant. Cost of the anti-malarial drug was significantly higher for artesunate-treated patients than for quinine-treated patients, whereas cost of hospitalization was significantly lower for artesunate-treated patients than for quinine-treated patients. Incremental analysis of ARTES against QLD as a baseline resulted in an ICER of $46.8/PRR_24_ and suggests ARTES as the most cost effective of all four treatment options.

**Conclusion:**

Artesunate is a cost effective malaria treatment option relative to quinine alternatives with the lowest incremental cost per unit of effectiveness.

*Trial registration* clinicaltrials.gov identifier: NCT02563704. Registered 19 September 2015, retrospectively registered

## Background

The deadline for the Millennium Development goals has come and gone but not all developing countries have achieved the targeted reductions in key health indices and improvements in human development; for example, the 4th millennium development goal was to reduce child mortality by two-thirds between 1990 and 2015 [1]. In 1990, the Cameroonian under-five mortality was 138 death per 1000 live births, but decreased to 88 deaths per 1000 live births in 2015, which is above the 46 deaths per 1000 live births that were targeted [2]. The recently adopted United Nations Sustainable Development goals, specifically targets a further reduction of under-5-mortality to 25 deaths per 1000 live births by 2030. Cameroon is thus amongst the countries with least progress and substantial changes are needed to reach the goal of 25 deaths per 1000 live births by 2030 [3].

Malaria is one of the most common childhood diseases and a major obstacle for economic and human development in sub-Saharan Africa. It is a leading cause of child mortality and constitutes the main cause of inpatient admission in paediatric wards. In 2011 in Cameroon, malaria was responsible for 24% of total deaths, 40–45% of medical consultations and 30% of hospital admissions in under-fives [4]. Effective case management of severe malaria can help reduce this mortality; however, a possible barrier to accessing appropriate case management is the cost of treatment [5].

The World Health Organisation (WHO) currently recommends parenteral artesunate as the drug of choice in the treatment of severe malaria in children and quinine as the second line drug [6, 7]. This recommendation is based on two key studies: SEAQUAMAT (South-East Asian Quinine Artesunate Malaria Trial) that was conducted in four South-East Asian countries with over 1461 patients (including 202 children) [8] and AQUAMAT (Artesunate versus Quinine in the treatment of severe malaria in African children) that was undertaken in 11 centres in 9 African countries and conducted with over 5400 children [9]. These studies revealed a relative reduction of mortality of 34.7 and 22.5% respectively in artesunate recipients. The costs of inpatient care of children with severe malaria were assessed in four of the 11 sites included in the AQUAMAT study. Overall, treatment with quinine was evaluated at US$ 63.5 and that with artesunate was evaluated at US$ 66.5 [10].

A review of policy by the Cameroon National Malaria Control Programme in 2013 recommended parenteral artesunate as first-line drug for treatment of severe malaria. The policy also recommended two regimens of quinine: a loading dose regimen of 16.6 mg/kg body weight quinine base (QB) as loading dose followed 8 h later by an 8-hourly maintenance dose of 8.3 mg/kg body weight; and a non-loading dose regimen of 8.3 mg/kg body weight QB every 8 h [11]. Some health institutions in Cameroon also use a twice daily administration of quinine at a dose of 12.5 mg/kg body weight QB every 12 h because of its feasibility based on limited personnel and 12 hourly nursing rotation. Although policy has been established, cost may still be a major factor limiting access to treatment for many patients. Monthly malaria expenditures as a proportion of monthly non-food household expenditures could be as high as 7.1 and 5.0% for rural and urban dwellers respectively [12]. This high economic burden of malaria can result in catastrophic costs and thus prevent people from seeking care when needed.

Cameroon is a lower middle-income country with a per capita income of $1407 and total health spending per capita of $59 per annum [13]. Although government spending has increased over a decade from $31 in 2002 to $59 in 2014, households continue to bear the greater part of the financial burden of healthcare. This is in spite of the fact that government provides free and heavily subsidised healthcare services covering treatment of malaria for children below 5 years of age. There are community based health insurance schemes serving various segments of the population but there is yet to evolve a common national strategy for Universal Health Insurance in Cameroon. Without the financial protection of a pre-paid health financing system, patients have to pay hospital charges for support services and purchase pharmaceuticals and consumables for the treatment of malaria. Therefore, more than 70% of the total health spending is still in the form of inequitable OOPs to supplement services in public facilities or to obtain care from the parallel private sector [14]. These funds come from financially constrained individuals and households. Cost is, therefore, a key issue in determining who can access appropriate and qualitative treatment of life threatening severe malaria.

Another factor determining the choice of anti-malaria treatment prescribed to patients is evidence-based knowledge on relative cost-effectiveness of treatment alternatives available to physicians and other key decision-makers in the health system. The healthcare market has unique features when compared to the market for normal goods where consumers are decision makers on choice and quantity of goods consumed. The agency relationship is a unique feature of healthcare in which the healthcare provider (supplier) rather than the patient (consumer) is the decision maker for choice of treatment alternative and indirectly determines the cost of health services consumed by patients. Such decision making by physicians is best supported by information from a structured comparative analysis or economic evaluation of costs and benefits of treatment alternatives.

Economic evaluations are increasingly being conducted alongside randomized comparative trials (RCT) as a means of providing researchers with individual patient data used to estimate cost effectiveness. Thus, a RCT of four different treatment alternatives for severe malaria was conducted—parenteral artesunate with three quinine regimens—using parasite reduction rate 24 h after onset of treatment (PRR_24_) as the main outcome measure and economic data collected from a healthcare perspective; in a hospital located in a malaria endemic geographical region of Cameroon: The Ebolowa Regional Hospital (ERH). Such an analysis of the costs and effectiveness of various treatment options for severe malaria will provide information underpinning government policymaking on financing malaria and paying hospital charges in resource constrained areas.

## Methods

### Study area

This study was conducted in the paediatric unit of ERH, which is the referral hospital in Ebolowa region of southern Cameroon. It has a total capacity of 158 beds for an estimated population of 120,000 inhabitants. The paediatric unit itself has a capacity of 28 beds and is headed by a paediatrician assisted by a physician intern, supported by ten nurses working in teams of 2–3 per shift. There are prescribed fees according to a government tariff for consultation, laboratory investigations and pharmaceuticals. These fees are payable in public health facilities including Ebolowa General Hospital where this study was carried out. The Ebolowa health district in which the hospital is located is a heavily malaria-endemic area characterized by a stable, perennial malaria transmission. Although the Ebolowa region of Cameroon is the headquarters of the South Region of Cameroon, it is less affluent than major cities, such as Douala and Yaoundé, the economic and political capitals respectively of Cameroon. The people are mainly farmers and small-scale traders with limited resources who will need to make rational decisions about utilization of healthcare facilities and treatment options.

### Study design

This economic evaluation was designed as a randomized comparative study of four treatment regimens, carried out from September 1st 2013 to March 31st 2014. Economic data was collected alongside clinical effectiveness data from each patient using a questionnaire administered by trained researchers. The result of the comparative effectiveness of the treatment alternatives has been previously reported and published [15].

### Study population

A consecutive sampling of all children admitted for severe malaria, who fulfilled to the inclusion criteria in the paediatric unit of the Ebolowa Regional Hospital during the study period was done. Inclusion criteria for the study were:

- Children with malaria parasitaemia positive for *Plasmodium falciparum* and confirmed on microscopy.
- Children aged 3 months to 15 years old irrespective of sex.
- Children presenting with one or more signs of severe malaria according to the 2013 Cameroon National Malaria Control Programme adopted criteria (impaired consciousness, abnormal behaviour, convulsions, prostration, persistent vomiting, jaundice, hyperthermia (temperature ≥40 °C), acute respiratory distress, clinical acidosis, haemoglobinuria, cardiovascular shock, dehydration, abnormal bleeding, severe anaemia, renal impairment, hypoglycaemia and hyperparasitaemia).
- Other differential diagnosis of the presenting symptoms was excluded.
- Parents gave a written informed consent.

Excluded from the study were children who have reported prior side effects to artesunate or quinine administration, severely malnourished children, and those who had a concomitant infection.

### Randomization and masking

Eligible patients were randomly assigned to receive either parenteral artesunate or one of the three quinine regimens. Using Kendall and Babington Smith random number table [16], an assistant not involved in the study performed the randomisation in advance in blocks of 20 composed of five of each regimen. Treatment allocations were placed in numbered opaque sealed envelopes to which the investigator was blinded till a patient was admitted. On admission, each patient was allocated an envelope corresponding to his unique identification number.

### Description of treatment alternatives and outcome measure

WHO policy on the case management of severe malaria recommends parenteral artesunate in prescribed doses as treatment of first choice; and where that is not available quinine can be used [6, 7]. Therefore, a comparison of parenteral artesunate with quinine in three different treatment regimens was chosen. The four treatment alternatives were:

1. Group 1 (ARTES) where patients received parenteral artesunate at 2.4 mg/kg at H_0_, H_12_, H_24_ for at least 24 h and then once daily till the patient could take oral drugs.
2. Group 2 (QLD) where patients received a loading dose of quinine base at 16.6 mg/kg followed 8 h later by an 8-hourly maintenance dose of 8.3 mg/kg quinine base for at least 24 h and then till the patient could take oral drugs.
3. Group 3 (QNLD3) where patients received 8.3 mg/kg quinine base every 8 h for at least 24 h and then till the patient could take oral drugs.
4. Group 4 (QNLD2) where patients received 12.5 mg/kg quinine base every 12 h for at least 24 h and then till the patient could take oral drugs.

Each patient was assessed at admission and admitted by the physician who developed a treatment plan. A consultant paediatrician would also review and approve the treatment plan including adjuvant treatment. Each dose of anti-malarial was administered by the nurse-on-duty and patient signs, symptoms and laboratory parameters were assessed at base case and daily until discharge. For each treatment regimen, parenteral treatment was relayed with an artemisinin-based combination (artemether–amodiaquine or artemether–lumefantrine) as soon as he could take oral treatment before being discharged from the hospital. The procedure for dilution and administration of the drugs and monitoring for clinical side-effects have been described in a previously published paper [15]. Malaria thick films were conducted on each patient at base case on admission and daily until discharge.

The outcome measure for an economic evaluation can be expressed in natural units or a generic measure, such as quality-adjusted life year (QALY) or disability-adjusted life year (DALY), based on expressed health preferences or in monetary units. For this study the primary outcome measure adopted for all treatment alternatives was in natural units—parasite reduction rate 24 h after onset of treatment (PRR_24_). A generic measure was not used because of the lack of locally generated preference based measures.

### Identification and measurement of costs

The study perspective is the viewpoint from which the costs and consequences of an intervention are evaluated. Although the societal perspective is the broadest perspective for an economic evaluation, costing for this study was conducted from the healthcare payer’s perspective. It is believed that this perspective covers the anticipated costs from treatment of severe malaria. Additionally, the lack of reliable data on productivity costs and market wage rates in developing countries made this the logical choice. The estimation covered direct medical costs attributable to each treatment alternative for severe malaria; namely the costs of anti-malarial drugs, nursing care materials, adjuvant treatment, laboratory investigations, hospitalisation and professional fees. Time and travel costs were excluded.

The time horizon refers to the length of time, over which costs and consequences are being evaluated. A suitable time horizon should be long enough to capture all associated costs and benefits of the treatment alternatives. The time horizon for this economic evaluation was over the course of the disease rather than the lifetime of the patient hence costs were identified, established and updated continuously from presentation at the facility, over the period of admission and ended at discharge. It was stopped at discharge because costs attributable to malaria or the treatment alternatives are negligible after discharge. The International Society for Pharmacoeconomics and Outcomes Research (ISPOR) guidelines were followed in the identification and measurement of all costs [17].

### Valuation of costs

The cost headings identified were in six categories as detailed in Table 1. In terms of valuation, the cost of anti-malarial drugs was determined using the price indicator guide obtained from the hospital pharmacy. If a drug was bought from a commercial pharmacy in town, a weighted price from a survey of sellers’ prices was used in the estimation. Unit prices of all other resources used in the analysis were obtained from two sources: the price/fee schedule of the Ebolowa Regional Hospital and commercial costs such as over-the-counter medications were determined by imputation from a survey of pharmaceutical vendors. Professional fees for doctor consultation and nursing time were determined by imputation from Cameroon Health sector salary scale. Laboratory tests included a suite of 3–15 thick blood films per patient and the cost of all these blood films was included in the price estimation even though the patient was billed for only one film. Total cost of hospitalization was obtained by multiplying the cost per inpatient day with the length of stay in the hospital.

**Table 1:** Overall cost per PRR_24_ and patient cost per PRR_24_

	ARTES	QLD	QNLD3	QNLD2
PRR_24_ (%)	92	74.8	66.5	71.7
Overall cost	1954.08	1747.12	1991.68	1807.14
Overall cost/PRR_24_	$21.24	$23.34	$29.95	$25.20
Mean cost per patient	65.14 (57.68–72.60)	62.40 (52.67–72.13)	66.40 (57.03–75.77)	60.23 (51.55–68.91)
Mean cost per patient/PRR_24_ (95% CI)	$0.71 (0.63–0.79)	$0.83 (0.7–0.96)	$0.99 (0.85–1.13)	$0.85 (0.73–0.97)

Economic studies of this nature typically consider costs rather than charges to reflect the opportunity cost of the alternative treatment option forgone. In many developing countries hospital charges may deviate from actual costs because of government subsidies. There was no means of converting these hospital charges and market prices and there is not an applicable cost-charge ratio. The pragmatic approach for this study was, therefore, to use the market prices as proxy for the opportunity cost. For same reasons a cost-to-charge ratio was not applied neither did we attempt to assess the impact of government subsidies on the hospital prices. There was no objective means of adjusting the prices based on these considerations and the difference in cost might not bias the study. Fixed costs such as hospital capital and overhead costs were considered to be costs common to all treatment alternatives. Additionally, such detailed costing data was not available at the Ebolowa District Hospital. Fixed costs were, therefore, not included in the analysis even though this could be a possible source of bias. All costs were initially expressed in CFA Francs in the base case analysis and then converted to US dollars (2016) in the final analysis. Although both clinical and economic data was collected, the paper on the clinical effectiveness was the more pragmatic and first to be published. It has taken some time to find requisite skills to develop and write the economic evaluation hence the time lag between data collection and publication. Since all costs were collected within a 12-month time frame, discounting of costs was not necessary.

### Cost effectiveness analysis

A cost effectiveness analysis (CEA) was chosen because it provides a framework to do a comparative analysis of the relative costs and effectiveness of all four alternative treatments. As a tool of economic analysis, it enables us determine the total cost of each alternative, the variance between the total cost and the separate cost headings and compare this with the effectiveness of each treatment alternative. Within this analysis, it was also possible to apply tests of significance to further confirm that variances in costs are not due to random differences. Finally, the costs and benefits of each alternative were compared using the mean cost per patient as a measure of cost and the parasite reduction in 24 h (PRR_24_) as a measure of effectiveness. The results are presented as cost per PRR_24_ as a measure of cost-effectiveness.

The incremental cost-effectiveness ratio is (ICER) is the additional cost that a decision maker has to bear in order to achieve an additional unit of effectiveness—in this case the cost of each additional measure of parasite reduction. The ICERs was calculated for all treatment options and the decision rules were applied. As there are no locally specified protocols for reporting economic evaluations, the Consolidated Health Economic Evaluation Reporting Standards based on the ISPOR Task Force Report of 2015 were followed [18].

### Statistical analysis

Data were coded as variables, introduced in Epi Info™ version 3.5.3 software and double-checked before analysis. In cases of patients with incomplete cost data, the missing information was estimated by imputation. Numerical variables were summarised into means and standard deviations. Categorical variables were summarised in cross-tables and Chi square test was used to compare the different proportions. A two-group comparison (ARTES/QLD, ARTES/QNLD3 and ARTES/QNLD2) of the different costs was done and Student *t* test was used to compare the mean costs in the different groups. Analysis was done by intention-to-treat and a p value less than 0.05 was considered statistically significant.

Both mean and incremental analysis of the costs was conducted. Mean costs were calculated for all cost headings while incremental analysis was carried out for the total cost of each treatment alternative. The median costs and confidence intervals are also reported. Although costs and effect of malaria fall within a very short time frame and its treatment pathway is limited, a simple probabilistic modelling in form of a decision tree was done, as well as calculations of the probabilities of each pathway, expected cost and expected effect. All treatment cycles ended in less than a month and therefore there was no justification for a Markov model.

## Results

**Fig. 1:**
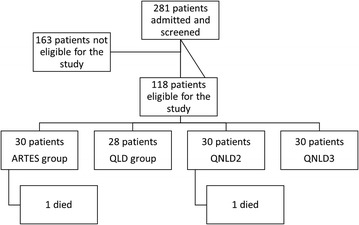
Study profile

A total of 281 children admitted for severe malaria at ERH were assessed for eligibility. Following diligent scrutiny of the inclusion and exclusion criteria, 118 patients were eligible and enrolled in the study. A total of 30 subjects were randomized to the ARTES (Artesunate) group, 28 subjects to QLD group, 30 subjects to QNLD3 group, and 30 subjects to QNLD2 group (Fig. 1). Two patients died, one in the ARTES group 4 h after onset of treatment and one in the QNLD2 group 18 h after onset of treatment, representing a mortality rate of 3.3% in each group. Consequently, data was analysed by intention-to-treat. The full analysis of resources used under each of the six cost headings by specification, measurement, valuation and source of data is detailed in Table 1.

### Clinical effectiveness

The results of the clinical effectiveness of each treatment group measured in PRR_24_, is presented in Table 1 and have been previously reported in another publication [15]. Results show that ARTES had statistically significantly higher effectiveness than any of the quinine treatment alternatives. ARTES recorded parasitological effectiveness of 92% followed by QLD with 74.8% and QNLD2 with 71.7%. The lowest parasitological effectiveness was recorded by QNLD3 with a PRR_24_ of 66.5%. In terms of secondary clinical outcomes such as fever clearance time and parasite clearance time were significantly shorter for artesunate-treated patients than for quinine-treated patients.

### Cost of antimalarial treatment

Two anti-malarial drugs—artesunate and quinine—were used in the study; details of specifications, quantities and costs are presented in Tables 2 and 3. During the study period, 93 ampoules of artesunate were bought against 48 ampoules of quinine for QLD group, 52 ampoules of quinine for QNLD3 group and 56 ampoules of quinine for QNLD2 group. The total cost of anti-malarial drug in ARTES group was 231.57 USD with a mean cost per patient of 7.72 USD which was significantly higher than that of the quinine groups (p < 0.001, see Tables 2, 5). In terms of proportion, anti-malarial represented 11.85% of total cost of treatment in the ARTES group compared to 3.87, 4.22 and 5.0% in the QLD, QNLD3 and QNLD2 groups, respectively; or an average of 4.36% in the quinine groups.

**Table 2:** Resource use by category, specification, measure, source and cost in CFA and USD

Resource category	Resource specification	Unit	Unit costs (CFA)	Unit cost (USD)^a^	Source of cost
A. Anti-malaria drug	Artesunate i/v 60 mg	Ampoule of 2 ml	1500	2.49	HPI/commercial survey
	Quinine i/v 1500 mg	Ampoule of 2 ml	850	1.41	HPI/commercial survey
B. Nursing care materials	Cleaning alcohol	1 pack of 50 ml	400	0.66	HPI/commercial survey
	Cotton	1 pack of 20 g	325	0.54	HPI
	Non-sterile gloves	1 pack of 2 units	100	0.17	HPI
	Infusion set	1 unit	175	0.29	HPI
	Intravenous catheter	1 unit	400	0.66	HPI
	Syringes	1 unit of 10 cc	55	0.09	HPI
C. Laboratory investigation	Thick film	Cost per test	1300	2.16	HPI
	Packed cell volume	Cost per test	1550	2.57	HPI
	Full blood count	Cost per test profile	4000	6.64	HPI
	Blood group, rhesus typing	Cost per test	2350	3.90	HPI
	CSF analysis	Cost per test	11,000	18.28	HPI
	Chest X-ray	Cost per film	5400	8.97	HPI
D. Hospitalisation	Admission fees	Cost per day	1500	2.49	HPI
E. Professional fees	Doctor consultation—GP	Cost per consultation	600	1.00	Hospital fee schedule
	Doctor consultation—paediatrician	Cost per consultation	2000	3.23	Hospital fee schedule
	Nursing care time	Cost per day	1500	2.49	Hospital fee schedule
	Out-patient consultation	Cost per patient	600	1.00	Hospital fee schedule
F. Adjuvant treatment	i/v fluids—dextrose	Pack of 500 mls	925	1.54	HPI/commercial survey
	i/v fluids—electrolytes	Pack of 500 mls	90	0.15	HPI/commercial survey
	Paracetamol injection	Ampoules of 1 g	2520	4.19	HPI/commercial survey
	Diazepam injection	Ampoules of 10 mg	170	0.28	HPI
	Phenobarb injection	Ampoules of 200 mg	325	0.54	HPI
	Furosemide injection	Ampoules of 10 mg	100	0.17	HPI
	Transfusion set	1 unit	275	0.46	HPI
	Transfusion fees	Cost per transfusion	15,000	24.91	HPI
	Naso-gastric tube	1 unit	2250	3.74	HPI
	Naso-gastric tube posing fee	Cost per insertion	2500	4.15	HPI
	Syringe	1 unit of 60 ml	3000	9.97	HPI

^a^Based on a currency conversion rate of 60,179CFA to $1 as at 8th January 2016

**Table 3:** Quantities and costs of antimalarial drugs by treatment alternatives

Description	ARTES n = 30	QLD n = 28	QNLD3 n = 30	QNLD2 n = 30
Number of antimalarial ampoules used	93	48	52	56
Mean number of ampoules per patient	3.1	1.71	1.73	1.87
Cost per ampoule—artesunate ($)	2.49	NA	NA	NA
Cost per ampoule—quinine ($)	NA	1.41	1.41	1.41
Total cost of anti-malaria treatment per group ($)	231.57	67.68	73.32	78.96
Mean cost per patient ($) (95% CI)	7.72 (7.28–8.16)	2.42 (2.07–2.77)	2.44 (2.05–2.83)	2.63 (2.05–3.21)

*NA* not applicable

### Cost of nursing care materials

The cost of nursing care materials in all patients ranged from 2.92 to 4.30 USD with a mean of 3.46 USD. The average cost of nursing care materials per patient was similar for the four groups (Tables 4, 5). Cost of nursing care materials as a proportion of total cost of treatment ranged from 5.3% in ARTES to 6.52% in the QNLD2 group.

**Table 4:** Quantities and cost of nursing care materials by treatment alternatives in USD

Nursing care materials	Unit cost	ARTES (n = 30)		QLD (n = 28)		QNLD3 (n = 30)		QNLD2 (n = 30)	
		Quantity	Total cost	Quantity	Total cost	Quantity	Total cost	Quantity	Total cost
Adhesive plaster (1 roll)	0.42	30	12.6	28	11.76	30	12.60	30	12.60
Alcohol (50 ml)	0.66	30	19.8	28	18.48	30	19.80	30	19.80
Cotton (1 packet)	0.54	30	16.2	28	15.12	30	16.20	30	16.20
Non sterile gloves (1 pair)	0.17	90	15.3	87	14.79	95	16.15	85	14.45
Infusion set (1 unit)	0.29	30	8.7	28	8.12	30	8.70	30	8.70
Intravenous catheter G24	0.66	30	19.8	28	18.48	30	19.80	30	19.80
Syringes 10 cc	0.09	123	11.07	118	10.62	129	11.61	124	11.16
Total cost per treatment group			103.47		97.37		104.86		102.71
Mean cost per patient in USD (95% CI)			3.45 (3.42–3.47)		3.48 (3.42–3.54)		3.50 (3.40–3.58)		3.42 (3.36–3.48)

**Table 5:** Summary of total cost in USD by cost headings according to treatment alternatives

Cost headings	ARTES (n = 30)		QLD (n = 28)			QNLD3 (n = 30)			QNLD2 (n = 30)		
	Cost per group	Cost per patient	Cost per group	Cost per patient	p value	Cost per group	Cost per patient	p value	Cost per group	Cost per patient	p value
Anti-malaria drugs	231.57	7.72	67.68	2.42	*<0.001*	73.32	2.44	*<0.001*	78.96	2.63	*<0.001*
Nursing care materials	103.47	3.50	97.37	3.477	0.49	104.86	3.50	0.44	102.71	3.42	0.61
Adjuvant care materials	619.24	20.64	560.56	20.02	0.48	566.95	18.90	0.57	498.61	16.62	0.99
Laboratory Investigations	635.25	21.17	620.74	22.16	0.58	798.87	26.63	0.17	726.85	24.23	0.53
Admission	147.50	4.92	170	6.07	*0.03*	192.5	6.42	*0.005*	167.5	5.58	*0.02*
Professional fees	217.05	7.24	230.77	8.24	0.33	255.18	8.51	0.25	232.51	7.75	0.57
Total and mean cost per treatment alternative (95% CI)	1954.08	65.14 (57.68–72.60)	1747.12	62.40 (52.67–72.13)	0.21	1991.68	66.40 (57.03–75.77)	0.67	1807.14	60.23 (51.55–68.91)	0.24

### Cost of adjuvant treatment

Mean cost of adjuvant treatment was 19.03 USD with a range of 3.52–51.31 USD. This cost heading represented 31.66–32.65% of the total cost of treatment in the various treatment alternatives. The total cost of adjuvant treatment was highest in the ARTES group with 619.24 USD but the difference with the other groups was not statistically significant (see Tables 5, 6).

**Table 6:** Quantities and cost of adjuvant treatment and materials in USD by treatment alternatives

Adjuvant treatment	Unit cost	ARTES (n = 30)		QLD (n = 28)		QNLD3 (n = 30)		QNLD2 (n = 30)	
		Quantity	Total cost	Quantity	Total cost	Quantity	Total cost	Quantity	Total cost
Dextrose solution (500 ml)	1.53	51	78.03	69	105.57	88	134.64	91	139.23
Electrolytes (500 ml)	0.15	101	15.15	102	15.53	107	16.05	112	16.80
Paracetamol injection (2 ml)	4.18	27	112.86	25	104.50	28	117.04	26	108.68
Diazepam injection (2 ml)	0.28	6	1.68	6	1.68	11	3.08	8	2.24
Phenobarbital injection (2 ml)	0.54	5	2.70	2	1.08	4	2.16	3	1.62
Furosemide injection (2 ml)	0.17	15	2.55	12	2.04	10	1.70	8	1.36
Transfusion set (1 unit)	0.46	15	6.90	12	5.52	10	4.60	8	3.68
Transfusion fees per pint of blood	24.91	15	373.65	12	298.92	10	249.10	8	199.28
Nasogastric tube	3.73	2	7.46	2	7.46	3	11.19	2	7.46
Nasogastric tube positioning fees	4.15	2	8.30	2	8.30	3	12.45	2	8.30
Syringe 60 cc, mean	2.49	2	9.96	2	9.96	3	14.94	2	9.96
Total cost per group			619.24		560.56		566.95		498.61
Median cost per patient (IQR)			26.31 (7.71–33.26)		9.40 (7.71–33.41)		9.46 (7.99–33.26)		9.60 (8.25–29.07)

Artesunate-treated patients used less dextrose solutions than quinine-treated patients (Table 6). The transfusion related costs in each group were also isolated. There were more transfusions in the ARTES group (15 compared to 8 in QNLD2) due to randomization, resulting in higher adjuvant costs than in any of the quinine groups. The additional transfusion costs were 383.10 USD in ARTES compared to 204.32 USD in QNLD2. When transfusion costs are deducted from the total cost of adjuvant treatment, QNLD3 has the highest adjuvant cost while ARTES has the lowest cost per patient (see Tables 6, 7).

**Table 7:** Transfusion related costs by the treatment groups (furosemide, transfusion set and transfusion fees)

	ARTES (n = 30)	QLD (n = 28)	QNLD3 (n = 30)	QNLD2 (n = 30)	Comments
TC (+ Tx costs) per group	619.24	560.56	566.95	498.61	ARTES has highest adjuvant care cost
Tx related costs	383.10	306.48	255.40	204.32	ARTES has highest transfusion cost
TC (− Tx costs)	236.14	254.08	311.55	294.29	
AC (+ Tx costs)	20.64	20.02	18.90	16.62	ARTES has lowest and QNLD3 has highest cost without Tx
AC (− Tx costs)	7.87	9.07	10.39	9.81	

*TC* total cost, *Tx costs* transfusion-related costs, *AC* adjuvant costs

### Cost of laboratory investigations

Quantities and cost of laboratory investigations varied widely among the groups. Overall, costs of laboratory investigations ranged from 9.04 to 55.36 USD with a mean 23.56 USD. The mean cost per patient for laboratory investigations was lower for patients in the ARTES group than for those in the quinine groups but the difference was not statistically significant (Tables 5, 8). In the study, a total of 750 thick blood smears was done for the entire patient population (ranging 3–15/per patient). The overall cost of laboratory investigations was higher in the non-loading quinine treatments than ARTES and QLD. The number of thick blood films and total cost of tests for malaria was highest in the non-loading quinine treatments with 501.12 and 475.20 USD respectively. The ARTES group also recorded higher quantities of tests for packed cell volume, full blood count and blood group/rhesus typing.

**Table 8:** Quantities and cost of laboratory investigations in USD by treatment alternatives

Laboratory investigations	Cost per unit (USD)	ARTES (n = 30)		QLD (n = 28)		QNLD3 (n = 30)		QNLD2 (n = 30)	
		Quantity	Cost in USD	Quantity	Cost in USD	Quantity	Cost in USD	Quantity	Cost in USD
Thick blood films	2.16	140	302.40	158	341.28	232	501.12	220	475.2
Packed cell volume	2.57	38	97.66	25	64.25	27	69.39	28	71.96
Full blood count	6.64	17	112.88	13	86.32	12	79.68	10	66.40
Blood group and rhesus typing	3.90	15	58.50	12	46.80	10	39.00	8	31.20
CSF analysis	18.28	3	54.84	4	73.12	6	109.68	4	73.12
Chest x-ray	8.97	1	8.97	1	8.97	0	0	1	8.97
Total cost per group			635.25		620.74		798.87		726.85
Median cost per patient (IQR)			18.86 (15.70–26.34)		18.53 (14.33–26.01)		23.93 (17.45–32.57)		23.93 (15.29–28.50)

### Cost of hospitalization

Duration of hospitalisation ranged from 0 to 5 days. The mean number of days on admission was shorter in the ARTES group followed by the QNLD2 group, QLD group and finally QNLD3 group (Table 9). Consequently, patients in the ARTES group spent less for hospitalization as compared to those in the quinine groups. The highest total cost of hospitalization of 192.5 USD and mean cost per patient of 6.425 USD was recorded in the QNLD3 group. This difference was statistically significant (Table 9).

**Table 9:** Quantity and cost of hospitalisation in USD by treatment alternatives

	Cost per day (USD)	ARTES (n = 30)		QLD (n = 28)		QNLD3 (n = 30)		QNLD2 (n = 30)	
		No of days	Cost	No of days	Cost	No of days	Cost	No of days	Cost
Admission fees	2.50	59	147.5	68	170	77	192.5	67	167.5
Mean number of days per patient		1.97		2.43		2.57		2.23	
Range		0–4		1–5		2–5		0–4	

### Cost of professional fees

Professional fees were calculated based on cost of nursing care per day, cost of doctor consultation for general practitioner (GP) and doctor consultation for paediatrician. The cost of nursing time as well as the total cost of professional fees was highest in the QNLD3 with 255.18 USD or 8.51 USD per patient. The lowest cost of 217.05 USD or 7.24 USD per patient was recorded in the ARTES group (Table 10).

**Table 10:** Quantities and cost of professional fees in USD by treatment group

	Cost	ARTES (n = 30)		QLD (n = 28)		QNLD3 (n = 30)		QNLD2 (n = 30)	
		No of units	Cost	No of units	Cost	No of units	Cost	No of units	Cost
Nursing care time per day	2.49	59	146.91	68	169.32	77	191.73	67	166.83
Doctor consultation per visit—GP	1.00	12	12.00	13	13.00	15	15.00	14	14
Doctor consultation per visit—paediatrician	3.23	18	58.14	15	48.45	15	48.45	16	51.68
Total cost			217.05		230.77		255.18		232.51

Overall cost per patient for the ARTES group was 65.14 USD, which was higher than for the any of the quinine groups although the difference was not statistically significant (see Table 8). Of the quinine groups, total cost was lowest in the QNLD2 group and highest in the QLD group. With regards to cost headings, the ARTES group recorded highest cost of anti-malarial drugs and adjuvant care materials but lower cost of laboratory investigations, admission and professional fees than the quinine groups. Within the quinine groups, the cost of adjuvant care, laboratory investigations, admission and professional fees was highest in the QNLD3 group and lowest in the QNLD2 group. The two-by-two comparison of the ARTES group with each quinine group showed no statistical significant differences between the groups (Table 5).

### Incremental cost effectiveness ratio

The ICER is an estimation of the cost to be paid for an extra unit of effectiveness of the more effective but costlier intervention; in this case ARTES. To determine the ICER, all four mutually exclusive treatment options were first listed in order of increasing cost, revealing that QNLD3 was dominated and QNLD2 was extendedly dominated and excluded from the analysis. Based on decision rules, ARTES was identified as the most cost effective treatment option relative to QLD, with an incremental cost of $46.78 per PRR_24_ (Table 11).

**Table 11:** Calculation of incremental cost effectiveness ratio

Treatment alternative	Cost ($)	Effect (PRR_24_)	Incremental cost ($)	Incremental effect (PRR_24_)	ICER ($ per PRR_24_)	Comment
QLD	51.45	0.8384				Baseline (B)
QNLD2	52.88	0.7987	1.43	−0.0397	36.020	Extended dominated
ARTES	57.85	0.9752	4.97	0.1765	28.159	Comparator (A)
QNLD3	58.18	0.7543	0.33			Dominated

$$ICER = \frac{{{\text{Cost}} {\text{A}} - {\text{Cost}} {\text{B}}}}{{{\text{Effect A}} - {\text{Effect}} {\text{B}}}} = \frac{57.85 - 51.45}{0.9752 - 0.8384} = \frac{6.4}{0.1368} = \, 46.78\$ /{\text{PRR}}_{24}$$

## Discussion

The study reveals that ARTES has higher cost ($65.14) than any of the quinine treatment options explored ($52.49–$62.40), but ARTES also has the highest clinical effectiveness overall. However, there is no significant difference in the overall cost of treatment of severe malaria with artesunate compared to quinine. This result compares favourably to the mean costs from studies by Lubell et al. [10] who recorded $66.5 for ARTES and $63.5 for the quinine groups. Since the management of severe malaria is mainly hospital-based, the study focused only on costs from presentation in hospital until discharge, excluding household costs, the collection of which is resource-intensive. Studies by Lubell also excluded household costs. When individual cost headings are considered; significant differences exist in the cost of anti-malarials (in favour of quinine) and in the cost of hospitalization (in favour of artesunate). Further analysis shows that the cost per PRR_24_ was lower for the artesunate group than for the quinine groups. In all the groups, with regards to proportion of total cost, adjuvant care and laboratory materials were the major costs incurred by patients.

On average, artesunate-treated patients significantly spent more for anti-malarials than quinine-treated patients with mean cost of $7.72 per patient compared to $2.5 in the quinine groups. This observation is similar to that of Lubell et al. [10], who evaluated cost in the AQUAMAT study. They obtained a mean cost of US$ 3.3 for artesunate ampoules as compared to US$ 1.3 for quinine ampoules. This difference in cost is due to the fact that, firstly, artesunate is more expensive than quinine. Secondly, once artesunate has been reconstituted, it must be discarded within an hour whereas quinine can be conserved for later use. The mean cost per patient for quinine ampoules in the QLD group was lower than that of the non-loading dose regimens. This difference can be explained by the fact that clinical recovery and parasitaemia negation were faster in the loading dose group than in the non-loading dose groups, thus duration of hospitalization was shorter for patients of the QLD group than for those of the QNLD3 and QNLD2 groups.

Adjuvant treatment considerably increases the cost of severe malaria and constituted 34.1% of the overall cost. Considering the average quantities of dextrose solution and electrolytes, smaller quantities were used for artesunate-treated patients than for quinine-treated patients. This is because dextrose was used to keep the vein patent for artesunate-treated patients whereas each dose of quinine was diluted in dextrose. Lower quantities and costs of dextrose and electrolyte solutions for the ARTES group were recorded compared to any of the quinine groups. This observation is similar to that of Lubell et al. [10] who estimated cost of fluids at US$ 12.5 for artesunate as compared to US$ 13.5 for quinine. However, it is important to note that a keep-vein-open is not compulsory for the treatment with artesunate. Patients who might not need an IV line can just have an indwelling catheter, through which artesunate will be administered as a bolus.

In this study, the cost of all the thick blood films done (3–15 per patient) was included in the cost of laboratory investigations though practically, this is usually determined only once on admission. With this consideration, the overall cost of laboratory investigations was lower for artesunate-treated patients than for quinine-treated patients. The difference observed in our study is principally because fewer thick blood films were done for artesunate-treated patients as a result of the rapid parasite clearance time associated with this drug and quick clinical recovery. Whereas including only one thick blood film in the estimates (as this will be practically done), the mean cost for laboratory investigations falls to 13.3 USD for ARTES group, 12.2 USD for QLD group, 12.1 USD for QNLD3 group and 10.6 USD for QNLD2 group. The mean cost is higher in the ARTES group because in the course of randomisation this group found itself with the highest number of patients with severe anaemia and a control haematocrit level was done to these patients on day 3.

The mean cost of hospitalization per patient was significantly lower for patients in the ARTES group than in the other groups. This difference can be explained by the fact that clinical recovery, parasite negation and hence duration of hospitalization was lower for artesunate-treated patients than for quinine-treated patients.

Cost of professional fees was calculated based on cost of nursing care per day, cost of doctor consultation for the GP and for the paediatrician. Cost of professional fees was highest in the QNLD3 group and the lowest cost was recorded in the ARTES group. This is due to the fact that administration of artesunate requires less nursing care and does not require any monitoring as compared to quinine, which requires monitoring of a rate-controlled infusion and thus nursing care is costlier. Moreover, clinical and parasitological recovery was longer for patients in the QNLD3 group, thus requiring more nursing care.

**Fig. 2:**
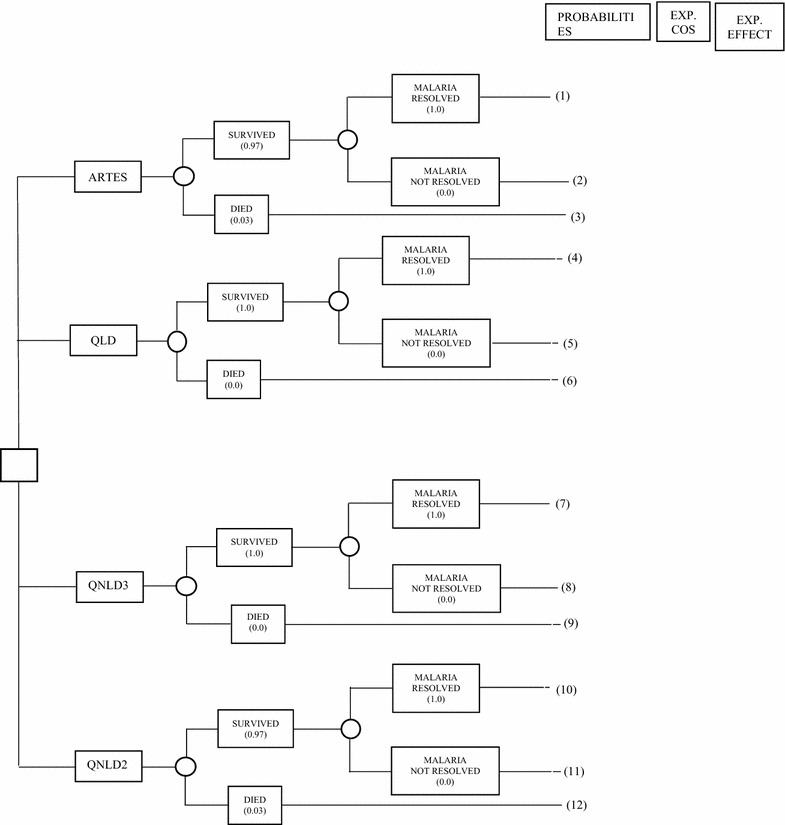
Probabilistic decision modeling for treatment options

Overall, treatment of severe malaria with artesunate had higher costs, but the difference was not statistically significant. Lubell et al. [10] obtained a similar result when comparing artesunate with the quinine loading dose regimen. As stated above, the overall cost of case management of severe malaria in this study included the cost of all the thick blood films done. Considering that this is usually done once on admission, the practical cost of treating severe malaria in this setting could be estimated at 50.01 USD for the ARTES group, 44.14 USD for the QLD group, 43.4 USD for QNLD3 group and 39.4 USD for QNLD2 group. Probabilistic modelling using the decision tree yielded expected cost of $63.18 in ARTES compared to $50.91 in QNLD2; however, ARTES recorded the lowest cost/effect because of the higher expected effect of 0.89 compared to 0.695 in QNLD2 (Fig. 2; Table 12).

**Table 12:** Probabilistic decision modelling: probabilities, expected cost and expected effect of treatment options

Treatment option	Pathway	Probabilities	Expected cost	Expected effect
ARTES	(1)	0.97	63.18	0.8900
	(2)	0.00	0.00	0.0000
	(3)	0.03	1.95	0.0276
			*65.13*	*0.9176*
QLD	(4)	1.00	62.40	0.748
	(5)	0.00	0.00	0.000
	(6)	0.00	0.00	0.000
			*62.40*	*0.748*
QNLD3	(7)	1.00	57.88	0.665
	(8)	0.00	0.00	0.000
	(9)	0.00	0.00	0.000
			*57.88*	*0.665*
QNLD2	(10)	0.97	50.913	0.695
	(11)	0.00	0.000	0.000
	(12)	0.03	1.576	0.022
			*52.488*	*0.717*

This cost can be related to the average standard of living in Cameroon. According to the 3rd Cameroon household survey in 2007, the standard of living of Cameroonians was estimated at 37.3 USD/head/month [19]. Hence, it can be inferred that treatment of malaria with artesunate at $65.14 is expensive because it costs more than the estimated monthly total expenditure; thus the need for treatment subsidy. It should be noted that the Cameroon government has subsidized malaria treatment in children less than 5 years old. In this age category and for pregnant women, treatment of severe malaria is free of charge [20, 21]. However, treatment of severe malaria remains expensive for the rest of the population. Moreover, this government subsidy only covers anti-malarials, dextrose solutions and nursing care materials; adjuvant treatment is not included. As shown in the results, adjuvant treatment considerably increased the cost of severe malaria (34.1% of the total cost). Thus subsidies can also target adjuvant treatment. The cost of ARTES can also be related to the decision threshold or maximum amount that policymakers are willing to spend to avert for an additional unit of effectiveness, usually expressed in life years (LY) or disability adjusted life years (DALY), estimated at $25 by the WHO. The measure of effectiveness was not LY or DALY but rather PRR_24_ from which was calculated the incremental cost per PRR_24_, making it difficult to compare the obtained ICER with existing decision thresholds.

## Limitations

The societal perspective of economic evaluation offers the most comprehensive framework for analysis but the study was conducted from a healthcare perspective because of the absence of benchmark productivity data. Furthermore, there are several methodological limitations relating to the choice of PRR_24_ over preference based measures such as QALY and DALY; inability to include fixed hospital costs in the analysis as well as the use of modelling and sensitivity analysis.

## Conclusion

The 3rd United Nations Sustainable Development Goals target to achieve access to qualitative healthcare services and access to safe, effective and affordable essential medicines on the platform of financial risk protection or universal health coverage can be undermined by lack of access to affordable malaria treatment.

The decision to adopt a drug of choice for severe malaria should, therefore, be based not only on clinical effectiveness, but also its effect on health budgets for individuals, households and governments. This study revealed that although artesunate is costlier than the quinine alternatives, it is more effective in terms of PRR_24_ and fever clearance. Cost effectiveness analysis suggests that artesunate is the most cost-effective of all treatment alternatives examined with an ICER of $46.78 relative to QLD. Nonetheless it remains expensive as relative to the standard of living of Cameroonians where most people live on an estimated $37 per month. Adoption of artesunate is best considered alongside some form of financial protection in the form of insurance schemes or treatment subsidies especially subsidies targeted at the cost of the artesunate and adjuvant therapy.
